# Repurposing FDA approved drugs against the human fungal pathogen, *Candida albicans*

**DOI:** 10.1186/s12941-015-0090-4

**Published:** 2015-06-09

**Authors:** Kevin Kim, Leeor Zilbermintz, Mikhail Martchenko

**Affiliations:** Keck Graduate Institute, Claremont, CA 91711 USA

**Keywords:** Antifungal, Drug-discovery, Off-label drug use, Small molecules

## Abstract

**Background:**

The high cost and prolonged timeline of new drug discovery and development are major roadblocks to creating therapies for infectious diseases. *Candida albicans* is an opportunistic fungal pathogen that is the most common cause of fatal fungal infections in humans and costs $2–4 billion dollars to treat in the US alone.

**Methods:**

To accelerate drug discovery, we screened a library of 1581 existing FDA approved drugs, as well as drugs approved abroad, for inhibitors of *C. albicans*. The screen was done on YPD yeast growth media as well as on the serum plate assay developed in this study.

**Results:**

We discovered that fifteen drugs, all which were originally approved for treating various infectious and non-infectious diseases, were able to kill *Candida albicans*. Additionally, one of those drugs, Octodrine, displays wide-spectrum anti-microbial activity. Compared to other selected anti-*Candida* drugs, Octodrine was shown to be one of the most effective drugs in killing serum-grown *Candida albicans* without significantly affecting the survival of host macrophages and skin cells.

**Conclusions:**

This approach is useful for the discovery of economically viable new therapies against infectious diseases.

**Electronic supplementary material:**

The online version of this article (doi:10.1186/s12941-015-0090-4) contains supplementary material, which is available to authorized users.

## Background

While almost all of us possess *Candida albicans* in our oral cavity, gastrointestinal tracts, genitourinary tracts, and on skin as a relatively harmless commensal organism, *C. albicans* is a major systemic fungal pathogen in humans [1]. *Candida* evades and escapes from the host’s innate immunity, causing irritating and recurrent infections that can range from thrush in immunocompetent colonized hosts, to life-threatening systemic infections in immunocompromised individuals such as patients with HIV, or those receiving immunesuppressing cancer chemotherapy and corticosteroids. Surprisingly, only 10 to 20% of individuals who develop bloodstream *Candida* infections are seriously immunocompromised. A large majority of patients develop *Candida* infections because they have become more susceptible while hospitalized due to the use of broad-spectrum antibiotics, surgery, and intravenous catheters. As a result, infections from *C. albicans* ranks as the fourth most common hospital-acquired infection in the United States. The cost of treating bloodstream *Candida* infections is $2–4 billion per year in the US alone [2]. In the US, annual incidence of systemic candidiasis is approximately 70,000 cases per year, which results in the death rate of about 30 to 40%, even after treatment with antifungal therapy [3].

The situation is especially grave in cancer patients. The incidence of *Candida* infection in all cancer patients is very high, ranging from 40 to 88 % [4, 5]. The mortality rate among the *Candida* infected cancer patients reaches an alarmingly high 75 % amongst the Filipino and Pacific Islanders [5]. Early antifungal treatment, although poorly tolerated by the host, is mandatory to improve the survival of cancer patients. Unfortunately, 30 % of *Candida* isolates are resistant to all antifungal treatments [6].

Host serum plays a prominent role in the pathogenicity of *C. albicans*. On the one hand, serum promotes morphological switching of *Candida* from yeast to hyphal forms, which is necessary for its evasion of phagocytosis and organ colonization [7]. On the other hand, serum is known to inhibit the activity of known antifungal drugs [8]. Taken together, these laboratory observations explain in part the clinical mortality observed during *Candida* blood infections, even when patients are treated with antifungals [5].

Several clinical and laboratory data suggests that currently available antifungal therapies are mostly ineffective in treating *Candida* infections [9]. Despite extensive research dedicated to the development of new therapeutic strategies, there are only a limited number of available drugs to fight against invasive fungal infections. Indeed, only four molecular classes targeting three distinct fungal metabolic pathways are currently used in clinical practice to treat systemic fungal infections. These include: fluoropyrimidine analogs, polyenes, azoles, and echinocandins [9, 10]. However, the efficacy of some of these drugs is severely limited because of their unacceptable toxicity, poor activity in blood, or the emergence of resistance; thereby underscoring an urgent necessity for new antifungal agents. Several other classes, such as morpholines and allylamines are only used as topical agents due to either their poor efficacy, or severe adverse effects when administered systemically [9, 10].

Unfortunately, the development of an entirely new drug is a long and expensive process. New drugs have to undergo an arduous approval process by the FDA in order to establish safety of the drug for human consumption [11]. We propose that the repurposing of existing FDA-approved drugs as antifungal agents may decrease the time and effort of bringing drugs with novel antifungal activity from the bench to the bedside. Recently, another group investigated the ability of FDA-approved drugs to inhibit *C. albicans* biofilm formation by screening the Prestwick Library, a commercially available chemical library of 1200 drugs [12]. However, *C. albicans* biofilm formation is just one of many pathogenesis strategies, such as yeast-to-hyphal phenotypic switching, white-opaque phenotypic switching, ability to adhere to mammalian tissues, and secretion of aspartyl proteinases [1, 7]. The goal of this study is to identify drugs capable of killing blood-borne *Candida albicans*, and we use serum as an *in vitro* surrogate of host blood. To this end, we have tested the anti-*Candida* activity of drugs from the Johns Hopkins Clinical Compound Library (JHCCL) [13] on *Candida* grown on serum-containing media. This library consists of drug-compounds that are FDA-approved with a diverse range of functions, mechanisms of action and well-characterized pharmacological and toxicological properties.

## Methods

### Candida albicans and bacterial strains

Strain SN250 is the wild type reference strain of *C. albicans*. It serves as the reference strain for our genetic knockout library screen. It is derived from the wild type strain SC5314, a human clinical isolate recovered from a patient with generalized candidiasis [14]. SN250 was used for drug screening experiments. The bacterial strains consisted of *Bacillus cereus* strain 10987 and *Escherichia coli* strain C600. The genetic screen for mutant sensitivity to Octodrine was tested with three *C. albicans* libraries that were previously created in [15–17].

### Media and growth conditions

*C. albicans* strains were cultured in liquid YPD medium at 30 °C overnight. *E. coli* and *B. cereus* were cultured in liquid LB medium at 37 °C and 30 °C, respectively, shaking overnight. A novel method to incorporate fetal bovine serum to agar was devised. Fetal bovine serum was preheated in a water bath set at 65 °C. We found that isothermal conditions of the two mixtures eliminated the formation of foam upon coalescence. The agar solution for the serum mixture consisted of 16 g agar, which was then brought up to 300 ml with nanopure water. The agar solution was then autoclaved at 120 °C for 45 min. The agar and serum mixtures were then amalgamated while in their isothermal states.

### Chemicals

An FDA-approved drug library comprising of 1500 drugs was purchased from Johns Hopkins, titled, Johns Hopkins Clinical Compound Library (JHCCL) version 1.0. The drugs arrived as 10 mM stock solutions in sealed microtiter plates and were made using DMSO or water as solvents. Drugs were arrayed in 96-well plates and screened at a stock concentration of 10 mM. Drugs were from Fisher and Sigma and were of the highest purity available. The library was stored at −20 °C until use. Prior to use, the library of drugs was thawed at room temperature. Drugs of interest were isolated and reproduced from prepared 10 mM solutions. Antimycin A, Captan, Chlorquinaldol, Clotrimazole, Disulfiram, Fluvastatin, Mycophenolic Acid, Methylbenzethonium Chloride, Miconazole, Nitroxoline, Octodrine, Pyrithione Zinc, Fluconazole, and Octanoic Acid were purchased from Sigma-Aldrich (St. Louis, MO, USA). Nifuroxime was purchased from MP Biochemicals (Solon, OH, USA). All drugs were prepared to 10 mM using DMSO as the solvent. DMSO was purchased from Amresco® (Solon, OH, USA). Fetal bovine serum (Triple Membrane 0.1 μm filtered) was purchased from GeneMate BioExpress (Kaysville, UT, USA).

### Screening assay

The sensitivity of *C. albicans* to drugs was assessed by a chemical screen. The absorbance at optical density at 600 nm (OD_600_) of yeast and bacterial overnight cultures were determined for each of our experiments. The absorbance values were then converted to cells/ml using McFarland’s scale. Twenty five million *Candida* cells were added to all 10 cm petri dishes; 600 million bacterial cells were added to all 10 cm bacterial petri dishes. Five microlitre of each drug was placed directly on the agar surface using a multichannel pipette and slight contact of the tip to the agar made to leave an impression to facilitate later analysis. Drugs that were replicated were done on petri dishes, following the same protocol in at least five independent experiments. For the replication studies, only a single drug was placed per plate. The plates were incubated at either 25 or 37 °C for 24 h. The drugs-of-interest were selected on their ability to produce a distinct zone of inhibition of fungal growth greater than the zone made by DMSO alone and at the same time that is comparable to, if not greater than the positive control, Fluconazole. The zones of inhibition were quantified by measuring their diameters in mm, as recommended by the Clinical Laboratory Standards Institute (CLSI) procedures outlined in the manual M44-A2 [18, 19]. In addition, we utilized the software ImageJ [20] to digitally quantify the magnitude of every zone of inhibition.

*E. coli* and *B. cereus* were cultured overnight in liquid LB media at 37 and 30 °C respectively. The sensitivity of *E. coli and B. cereus* to Octodrine was assessed by spreading 6 × 10^8^ cells of their respective overnight cultures onto petri dishes containing solid LB media. 5 μl of neat Octodrine was placed directly on the agar surfaces. The plates with *E. coli* were incubated at 37 °C, while plates with *B. cereus* were incubated at 30 °C for 24 h.

### Determination of minimal effective drug concentrations by drug diffusion susceptibility testing

Plates inoculated with *C. albicans* were prepared using the protocol described above. To elucidate the ability of the varying concentrations of drugs to form zones of inhibition, two-fold serial drug dilution experiments were performed. To perform the first two-fold dilution, one part of the 10 mM stock solution was mixed with one part DMSO. Each subsequent dilution was done with aliquots from the prior dilution mixed with equal parts of DMSO. 5 μl of each drug dilution was spotted onto a lawn grown on YPD plate, as well as 5 μl of DMSO as a negative control. Dilutions beyond 0.07813 mM that were still forming a significant zone of inhibition were further diluted on a separate plate. Drug-treated plates were then incubated at either 25 or 37 °C for 24 h. Two-fold serial dilutions of Octodrine were done on YPD and Serum plates with undiluted Octodrine as the most concentrated solution.

### Genotypic mutant screening against Octodrine

Each *C. albicans* knockout strain from one of the three *C. albicans* libraries [15–17] was cultured in individual wells of 96 well plate in 100 μl of YPD media overnight at 30 °C. 5 μl of each *Candida* knockout overnight culture was spotted onto a YPD solid plate using a multichannel pipette. The cells were then left to absorb into the YPD plates for 1 h at 25 °C. Using a multichannel pipette, 5 μl of Octodrine 10 mM was spotted directly on top of the *C. albicans* cells. The plates were then placed in an incubator set at 30 °C and left overnight. Analysis of plates consisted of isolating any strains that exhibited resistance to 5 μl of 10 mM Octodrine. Resistance was noted by the ability of the *Candida* cells and the subsequent drug-treated spot to lack a zone of inhibition.

### Mammalian cell culture, drug treatment, and survival assay

RAW264.7 mouse macrophage cells (ATCC number TIB-71) and human melanoma C32 cells (ATCC number CRL-1585) were maintained in DMEM (Sigma-Aldrich) supplemented with 10 % FBS (Bioexpress) and 100 μg/mL penicillin and 100 μg/mL streptomycin. Mammalian cells (10,000 per well) were seeded in 96-well plates (100 μl/well) 24 h before the assay. During the assay, 1 μl of 10 mM drug was added to 100 μl of cell-containing media. Two-fold serial dilutions of the media were performed. RAW264.7 and C32 cells were treated with drugs for 24 and 48 h respectively, and determination of cell viability by 3-(4,5-dimethylthiazol-2-yl)-2,5-diphenyltetrazolium bromide (MTT) assay was performed as described in [21]. Each data point shown in the figure for MTT assays represents the average of results from at least two wells in each of at least two separate experiments. Cell viability is shown as the percentage of survivors obtained relative to untreated cells grown in media only (100 %).

### Image capture and image processing

All images were taken with an 8MP iSight camera with an aperture size of F2.4 and touch-to-focus capabilities. Images were standardized with a universal template to allow for direct comparison between images. Images are stock and unaltered by any graphics editing software.

## Results

### Moderate ability of Fluconazole to kill serum-grown Candida albicans at 37 **°**C

**Fig. 1:**
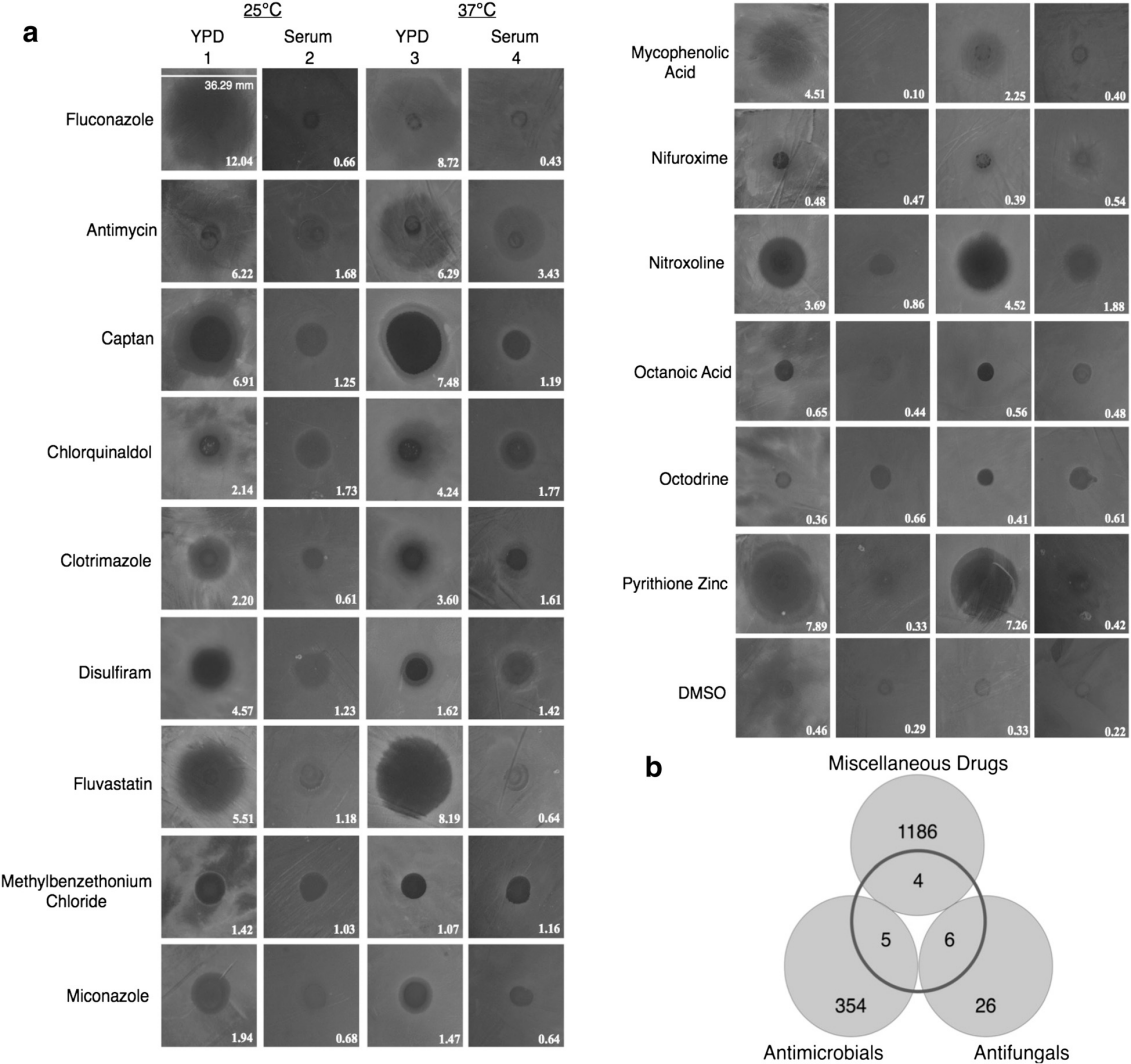
The search for new antifungal drugs. **a** Analysis of *C. albicans* strain SN250 treated with various drugs. The strains were grown and exposed to drugs determined to be drugs of interest by our chemical screen. The size of the zone of inhibition on each image is shown with the same scale (*mm*), with standard deviation values are shown in Additional file 1: Table S1. Plates were treated with drugs after spreading of 200 μl of liquid *Candida* culture and left to incubate overnight in either 25 or 37 °C. **b** The hits were classified into three classes: *Antifungal*, *Antimicrobial*, and *Miscellaneous drugs*. Numbers within *grey circles* represent number of drugs of each class in JHCCL, while numbers in the *transparent middle circle* shows the number of hits of drugs from our screen

It has been reported that host serum markedly inhibits growth of the human fungal pathogen, *Candida albicans* [22–24]. We have confirmed these observations by showing that *Candida* wild type strain SN250 [15] is unable to grow in liquid 100 % Fetal Bovine Serum at either 25 or 37 °C (data not shown). However, during the course of our experiments, we have observed that *Candida* is able to grow and establish a lawn on solid serum plates containing 62.5 % v/v FBS and 37.5 % v/v agar solution, at both temperatures (Fig. 1a).

We investigated whether Fluconazole, the widely used anti-*Candida* drug, was able to kill *C. albicans* on solid serum plates. While Fluconazole was shown to be an effective inhibitor of *Candida* growth on standard solid YPD plates, (Fig. 1a), we observed that Fluconazole moderately inhibits the growth of *Candida* on serum plates (Fig. 1a and Additional file 1: Table S1). It should be noted, however that Fluconazole formed prominent zone of inhibition on the solid YPD plates. In addition, we observed that Fluconazole exhibited diminished effectiveness at forming zone of inhibition at 37 °C compared to 25 °C (Fig. 1a and Additional file 1: Table S1). We decided to assess the drug-pathogen interactions at 25 °C in additional to body temperature because there is a serious problem of *Candida* growth in catheters where it may be exposed to serum at room temperatures [25].

### Screening of inhibitors of Candida albicans lawn formation

In light of the moderate effectiveness of Fluconzole to kill *C. albicans* in serum at the physiologically relevant temperature (37 °C), and in search for alternative anti-fungal drugs, we decided to screen the library of chemicals [13] approved by the FDA for human use for their ability to kill *Candida albicans*. JHCCL consists of 1581 FDA-approved drugs, as well as drugs approved abroad, consisting of small molecules (10 mM) that are used as treatments for a variety of diseases, including, but not limited to: infectious, neurodegenerative, psychiatric, cardiovascular diseases and cancer.

We identified inhibitors of *Candida* growth formation by performing a primary screen on the 1581 drugs belonging to the JHCCL. We plated *Candida* on solid YPD and serum plates, and placed 5 μl of each 10 mM drug on top of the fungal lawn. We tested the effect of exposure to each of the drugs from the library on their ability to inhibit *Candida* growth, as well as their ability to form a zone of inhibition within the fungal lawn. Based on this screen, we identified 15 drugs that inhibit *Candida* growth on solely YPD or on both YPD and serum plates in at least five independent experiments at two temperatures, 25 °C and 37 °C (Fig. 1a). We classified the 15 hits into three different classes to facilitate interpretation of our results. They were: 6 hits from 32 known antifungals, 5 hits from 359 antimicrobials/antiseptics, and 4 hits from 1190 other multifunctional drugs (Fig. 1b). While these drugs were chosen in these screens for their ability to inhibit *C. albicans* lawn formation, the actual levels of inhibition varied from weak to strong inhibition (Fig. 1a and Additional file 1: Table S1).

All 6 selected antifungal drugs, Antimycin A, Captan, Clotrimazole, Fluconazole, Miconazole, and Pyrithione Zinc showed strong inhibition of *Candida* growth on YPD plates at 25 °C and 37 °C (Fig. 1a and Additional file 1: Table S1). However, with the exception of the pesticide Captan, all of the antifungals exhibited weak inhibition of *Candida* growth on serum plates (Fig. 1a and Additional file 1: Table S1).

Out of the five antimicrobial/antiseptic drugs selected by our screen, Chlorquinaldol and Methyl-benzethonium chloride displayed the strongest inhibition of *C. albicans* growth on serum plates. Nifuroxime, Nitroxoline, and Octanoic acid showed weaker inhibition of *Candida* growth on serum plates (Fig. 1a and Additional file 1: Table S1).

Interestingly, from a drug-repurposing point of view, 4 drugs from 1190 of the other multifunctional drugs, showed anti-*Candida* activity on YPD plates (Fig. 1a and Additional file 1: Table S1). Of those, Fluvastatin and Mycophenolic acid showed very strong inhibition of YPD grown *Candida*, but failed to inhibit its growth on serum plates (Fig. 1a and Additional file 1: Table S1). The other 2 drugs, Disulfiram and Octodrine showed a consistent moderate-to-weak anti-*Candida* activity on YPD and serum plates (Fig. 1a and Additional file 1: Table S1).

### Diffusion susceptibility testing of the hits obtained from the screen

The 15 hits from the primary screen consisting of antifungals, antimicrobials, and miscellaneous drugs were tested in diffusion susceptibility assays in order to determine their potency against *C. albicans* lawns. These confirmatory screenings were performed over a range of drug concentrations, where 5 μl of drugs within the range of 10 mM to 0.3 μM were applied on YPD-grown *Candida* cells and plates were incubated for 24 h. YPD plates were chosen as the testing media since some of the drug hits were ineffective in inhibiting fungal growth on serum plates. The efficacy of each drug was evaluated by estimating the inhibitory concentration at which the drug formed a zone of inhibition on a fungal lawn of YPD plates.

**Fig. 2:**
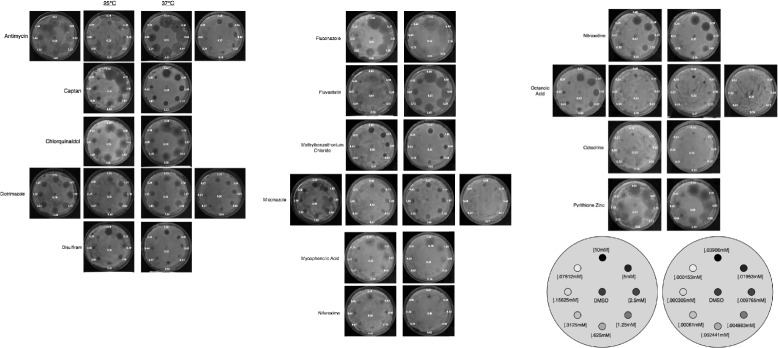
Agar drug diffusion susceptibility assay of *Candida albicans* SN250. Plates were treated with various concentrations of drugs after spreading of 200 μl of liquid *Candida* culture and left to incubate overnight in either 25 or 37 °C. The middle of the plate served as the negative control, DMSO. Dilutions that failed to exhibit discernable inhibition after one plates were further diluted a second time. The size of the zone of inhibition on each image is shown with the same scale (*mm*), with standard deviation values are shown in Additional file 1 Table S2

We found that with the exception of Fluconazole and Captan, all antifungals were able to inhibit *Candida* growth in the μM drug range (Fig. 2 and Additional file 1: Table S2). All five antimicrobial and antiseptic drugs were only able to inhibit fungal growth in the mM drug concentrations (Fig. 2 and Additional file 1: Table S2). Surprisingly, among the drugs approved for non-infectious disease treatments, Fluvastatin was able to inhibit *C. albicans* growth in the μM drug concentration, while the rest of the drugs were only inhibitory in the mM drug concentrations.

### Broad-spectrum antimicrobial properties of Octodrine

**Fig. 3:**
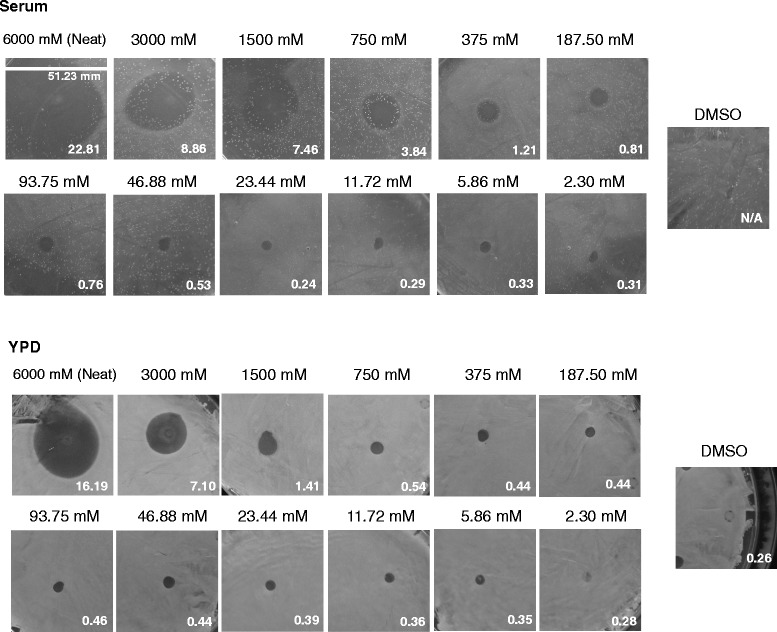
Agar Octodrine diffusion susceptibility assay of *Candida albicans* SN250. Plates were treated with various concentrations of Octodrine after spreading of liquid *Candida* culture and left to incubate overnight in either 25 or 37 °C. The equal volume of negative control, DMSO, was also included. The size of the zone of inhibition on each image is shown with the same scale (*mm*), with standard deviation values are shown in Additional file 1 Table S3

**Fig. 4:**
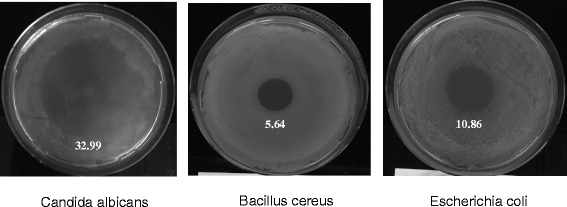
The sensitivities of *Candida albicans*, *Escherichia coli*, and *Bacillus cereus* to neat Octodrine. Plates were treated with 5 μl of undiluted Octodrine after spreading of 200 μl of liquid *Candida* (25 × 10^6^ cells) or bacterial cultures (600 × 10^6^ cells) on 10 cm petri dish and left to incubate overnight in either 37 °C for *C. albicans* and *E. coli* or in 30 °C for *B. cereus*. The size of the zone of inhibition on each image is shown with the same scale (*mm*), with standard deviation values are shown in Additional file 1 Table S4

Although nine antimicrobial and non-antimicrobial drugs we discovered have not been approved by FDA to treat fungal infections, eight of them were found previously to kill *Candida albicans* [26–39]. Octodrine, the drug previously used as a decongestant [40, 41], showed an ability to kill serum-grown *C. albicans* when applied at 10 mM 5 μl drop (Fig. 1). Furthermore, Octodrine is the only drug that has not been tested to kill fungi previously. We wanted to investigate whether the application of an even higher concentration of Octodrine would augment its antifungal properties. To test this, we applied 5 μl of neat, undiluted Octodrine, which is produced in a liquid form, as well as its two-fold serial dilutions on the serum and YPD grown *Candida* lawn. We discovered that the undiluted amount of Octodrine was effective in eliminating the fungal growth (Figs. 3 and 4 and Additional file 1: Tables S3 and S4). We also observed that Octodrine is more effective in inhibiting serum grown *Candida* compared to YPD grown *Candida*, as the inhibitory concentrations of Octodrine were more pronounced on serum than on YPD plates (Fig. 3, Additional file 1: Table S3).

In order to identify *Candida* proteins and signaling pathways that mediate the lethality of Octodrine, we screened three *C. albicans* knockout libraries, collectively consisting of 908 mutant strains lacking one of the previously demonstrated virulence genes, for any alterations in sensitivity to 10 mM Octodrine. These libraries consisted of 647 mutant strains lacking one of the essential virulence genes [15], 96 cell wall protein mutants [42], and 165 transcription factors mutants [43]. We found that all *Candida* mutants were as sensitive to 5 μl of 10 mM Octodrine as the wild type strain (Additional file 1 Figure S1).

We hypothesized that Octodrine may kill microorganisms by targeting their non-protein cellular components. To investigate this we tested the sensitivities of *Escherichia coli* strain C600 and *Bacillus cereus* strain 10987, gram-negative and gram-positive bacteria, respectively, and frequent disease-causing bacteria, to Octodrine. These bacterial strains were chosen because they are wild type strains with known genotypes and genomes [44, 45]. We observed that 5 μl of neat Octodrine formed a prominent zone of inhibition in both *E. coli* and *B. cereus* lawns (Fig. 4 and Additional file 1: Table S4), which suggests that Octodrine possesses wide spectrum antimicrobial properties.

Since Octodrine was one of the most prominent drugs that killed *C. albicans* in serum, we tested the sensitivity of mouse macrophage cell line RAW264.7 to Octodrine, and compared it to the rest of selected drugs. Since Octodrine stock was dissolved in DMSO, we had to test the sensitivity of the cell line to this solvent alone. Cell viability was determined by MTT assay (Methods) and was calculated as the percentage of surviving cells in various chemical concentrations relative to cells treated with media alone. We observed that the sensitivity of mouse macrophages to Octodrine was the close to the sensitivity to the DMSO alone (Table 1). This strongly suggests that Octodrine concentrations that kill *Candida albicans* in serum (Fig. 1a) do not affect the survival of host phagocytes. The sensitivity of RAW264.7 cells to DMSO has previously been reported [46], and is consistent with the cellular sensitivity seen here. We tested the sensitivity of RAW264.7 macrophage like cells because macrophages are first line of defense against *Candida*, and knowing the sensitivity of those cells to antifungal is an important question. We confirmed these observations by testing sensitivity of human skin melanoma cell line, C32 (Table 1). With the exception of Octodrine, Floconazole, Nifuroxime, and Fluvastatin, all other selected drugs adversely affected the survival of host cells, which suggests that they may have undesirable side effects when used in blood. We chose this cell line because of the relevance of skin to *Candida* infections.

**Table 1:** Minimal cytotoxic concentration (MCC) calculations for the cell lines RAW264.7 and C32 treated with selected drugs

Drug	MCC (μM) RAW264.7	MCC (μM) C32
Fluconazole	100.00	100.00
Antimycin	3.13	0.098
Captan	12.50	12.50
Chlorquinaldol	0.20	6.25
Clotrimazole	6.25	12.50
Disulfiram	12.50	12.50
Fluvastatin	50.00	6.25
Methylbenzethonium Chloride	6.25	1.56
Miconazole	6.25	1.56
Mycophenolic Acid	0.78	1.56
Nifuroxime	25.00	25.00
Nitroxoline	6.25	6.25
Octanoic Acid	0.78	6.25
Octodrine	25.00	25.00
Pyrithione Zinc	0.78	1.56
DMSO	50.00	50.00

The solvent DMSO is used as a negative control. Mammalian cells were treated with drugs at concentrations shown. The drugs were serially-diluted, and the MCC is defined as the first concentration of the drug, which is able to lower cell viability below 100 %. Cell viability was determined by MTT assay (Methods) and is shown as the percentage of survivors relative to cells treated with media alone

## Discussion

This study was designed to test FDA and foreign-approved small molecules drugs for their antifungal properties, with the objective of reducing the cost and time necessary to develop much needed anti-*Candida albicans* therapies. This library consists of an FDA-approved, off-patent collection of 1581 small molecules (10 mM) that are used as drugs for a variety of diseases, including infectious, neurodegenerative, psychiatric, cardiovascular diseases and cancer. Such an approach would rapidly expedite the drug discovery and development process since the general pharmacology, toxicology, and pharmacokinetic properties of all these drugs are already well established. This would facilitate the further analysis of the novel functionalities of the established molecules because the structure, chemical properties, and biological functions of almost all members of this library are known.

We have developed a serum-based assay to address the limitations of currently used YPD media, whose components do not represent *in vivo* components that support the growth of *Candida*. The moderate-to-mild effectiveness of Fluconazole and other FDA approved antifungal drugs on our serum assay is comparable to the effectiveness of Octodrine in killing serum-grown *C. albicans*.

**Fig. 5:**
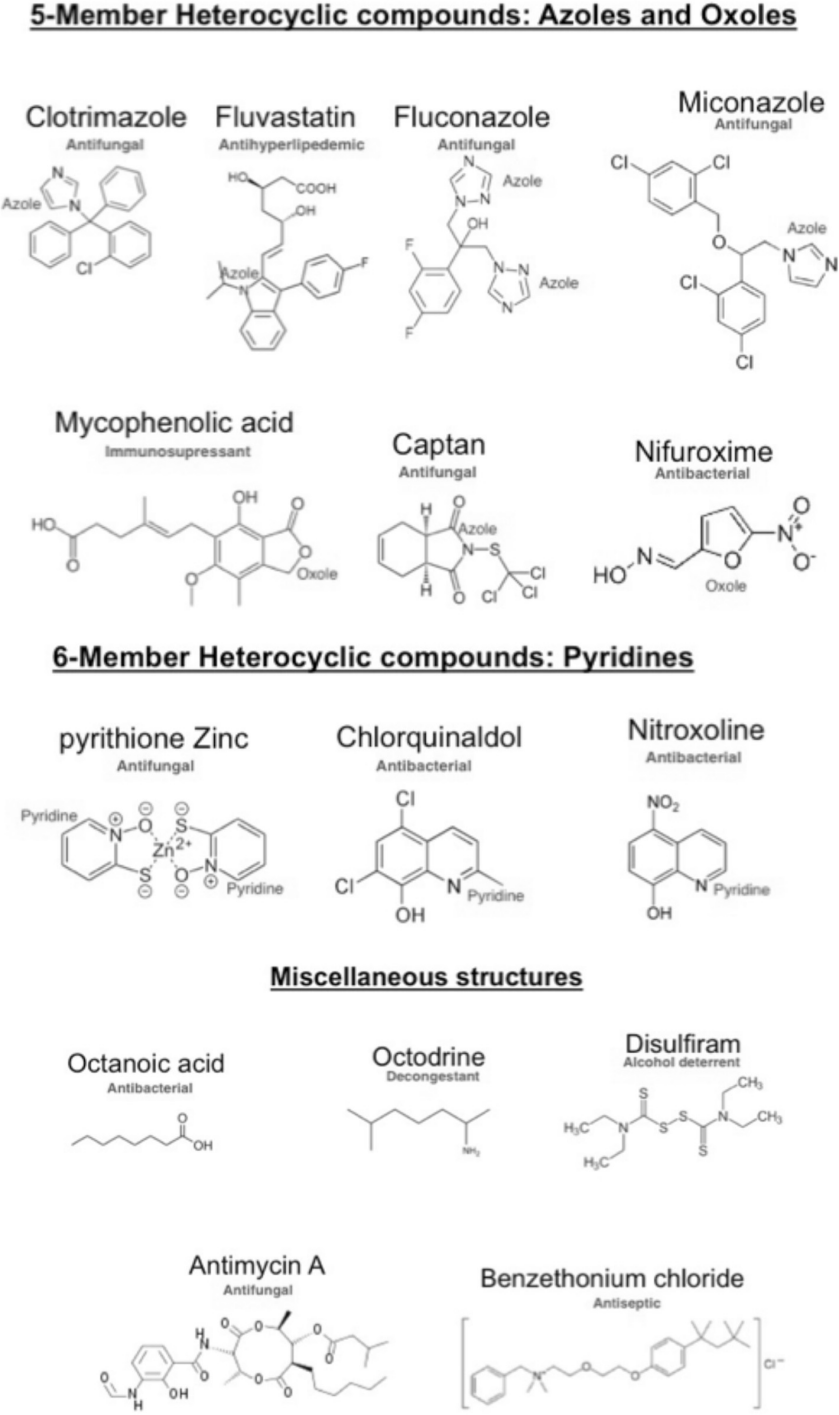
Chemical structures of 15 drugs shown to have anti-*Candida albicans* activity in our screen. All drugs were categorized into 3 classes: 5-membered heterocyclic (*Azoles and Oxoles*) and 6-membered heterocyclic (*Pyridines*) compounds, as well as drugs of other structures. The FDA approved applications are shown in *green*. Substructures are indicated in *red*

In our study, fifteen out of 1581 drugs displayed anti-*Candida* properties. Overall, all drugs discovered in our study could be separated into three structural categories: five-membered heterocyclic drugs, such as azoles and oxoles, six-membered heterocyclic compounds (pyridines), and other structures (Fig. 5). The fact that we selected six drugs that were previously approved by FDA to treat fungal infections biologically validates our approach. The six antifungal drugs obtained as hits from the screen comprised three different chemical classes: azoles (Fluconazole, Captan, Clotrimazole, and Miconazole), pyridine (Pyrithione Zinc), and other structures (Antimycin A). Additionally, we found five antimicrobial/antiseptic drugs to be effective at inhibiting *C. albicans* lawn formation in the screen. These drugs include general antiseptics and antibacterial antibiotics, and comprise three different chemical classes: oxoles (Nifuroxime), pyridines (Nitroxoline and Chlorquinaldole), and other structures (Octanoic acid and Benzethonium Chloride) (Fig. 5). The fact that these five other drugs that were approved by FDA to treat other non-fungal infectious diseases were also observed to kill *Candida albicans* shows that these drugs could be repurposed to be broad-spectrum anti-microbial drugs.

Lastly, from the point of view of drug repurposing, the most interesting class of drugs would be the one with demonstrated activity against a variety of diseases, but with no known or characterized antifungal activity to date. From the screen, we found four drugs belonging to this class to be effective in preventing *Candida* growth. These drugs have been designed for several indications, including immune-suppression (Mycophenolic acid), deterrent of alcohol consumption (Disulfiram), antihyperlipidemic (Fluvastatin), and decongestant (Octodrine). These drugs comprise three different chemical classes: azole (Fluvastatin), oxole (Mycophenolic acid), and other structures (Disulfiram and Octodrine) (Fig. 5). These four drugs that were approved to treat non-infectious diseases showed antifungal properties and thus, could be repurposed as new antifungal drugs.

Recently, another group investigated the ability of FDA-approved drugs to inhibit *C. albicans* biofilm formation [12] by screening the Prestwick Library, a commercially available chemical library of 1200 drugs. Interestingly, the authors of that paper discovered several antifungal drugs in common to our study: Miconazole, Clotrimazole, and Methylbenzonium chloride. The difference between the two studies is that Siles et al. [12] screened for drugs capable of inhibiting *Candida* biofilm formation, while we looked for FDA approved drugs capable of inhibiting the growth of non-biofilm *C. albicans* growth. In addition, with the exception of Octodrine, every one out of fifteen discovered drugs in our study had been previously tested for the ability to kill *Candida albicans* [26–39].

In this study, Octodrine, which was previously used as a decongestant and registered under the name of Vaporpac (Medley & James Laboratories) [40, 41], was a drug that had not been previously established as an anti-fungal. We observed that although its anti-*Candida* activity was mild on YPD, it displayed one of the best *Candida* growth inhibition on serum compared to other drugs. It also has a potential to be one of the safest of the discovered drugs because it did not affect the sensitivity of mammalian cells significantly. The fact that no *C. albicans* mutants showed a decrease in sensitivity to Octodrine argues against the potential emergence of Octodrine-resistant *Candida* strains, and favors the usage of this drug as a new antifungal treatment against *Candida*. In addition, we showed that Octodrine is capable of killing Gram-positive as well as Gram-negative bacteria, making it a desirable broad-spectrum antimicrobial countermeasure, that probably kills microbes by targeting their non-protein components. Octodrine was previously shown to agonize Estrogen Receptor Alpha with the potency of 21 μM [47]. Thus, any *in vivo* antimicrobial activity of Octodrine in humans would have to be in the nM range to avoid this side effect. As the pharmacokinetics of Octodrine had been previously established in numerous animal models [48], this approach is useful for the discovery of economically viable new therapies against infectious diseases.

## Conclusions

In summary, we have screened the Johns Hopkins Clinical Compound Library, a commercially available chemical library of FDA approved 1581 drugs, for the identification of bioactive drugs against *C. albicans* growth. Our results provide the comprehensive survey of the inhibition of *Candida* growth by existing drugs, one of which hadn’t been previously reported to have antifungal properties. From a drug repurposing point of view, the identification of drugs with no known antifungal activity and which demonstrated excellent activity against *C. albicans* growth in serum opens up a valuable new avenue for the rapid development of antifungal agents, which are urgently needed.

## Additional file

Additional file 1:
**Quantification of the zone of inhibition post-treatment of various drugs on** ***C. albicans*** **strain SN250.** Quantification of the zone of inhibition post-treatment of various serially diluted drugs on *C. albicans* strain SN250. Serial dilution experiments of Octodrine on serum and YPD plates. Quantification of the zone of inhibition of Octodrine in neat form against *Candida albicans*, *Escherichia coli and Bacillus cereus.* Elucidation of mechanism of *Candida albicans*: Elucidation of *Candida albicans* sensitivity to Octodrine.
