# Supplementary material for: Repurposing FDA approved drugs against the human fungal pathogen, Candida albicans
Source: Ann Clin Microbiol Antimicrob. 2015 Jun 9;14:32. doi: 10.1186/s12941-015-0090-4 (PMC4462072; doi:10.1186/s12941-015-0090-4)

Supplemental Table 1: Quantification of the zone of inhibition post-treatment of various drugs on *C. albicans* strain SN250.

A)

	YPD 25°C			YPD 37°C		
**Drug**	**ZOI Area (mm^2)**	**Number of Pixels**	**ZOI Area/ Number of Pixels**	**ZOI Area (mm^2)**	**Number of Pixels**	**ZOI Area/ Number of Pixels**
**Fluconazole**	933.84 ± 5.92	77.55 ± 0.21	12.04 ± 0.03	822.65 ± 5.42	94.35 ± 0.11	8.72 ± 0.01
**Antimycin**	507.27 ± 5.31	81.57 ± 0.34	6.22 ± 0.09	541.4 ± 4.34	86.02 ± 0.24	6.29 ± 0.02
**Captan**	512.19 ± 4.31	74.1 ± 0.23	6.93 ± 0.02	579.1 ± 5.09	77.46 ± 0.25	7.48 ± 0.02
**Chlorquinaldol**	194.72 ± 3.49	90.86 ± 0.22	2.14 ± 0.01	365.57 ± 4.54	86.19 ± 0.14	4.24 ± 0.01
**Clotrimazole**	210.77 ± 3.21	95.71 ± 0.23	2.20 ± 0.01	317.16 ± 3.42	88.2 ± 0.15	3.6 ± 0.01
**Disulfiram**	340.1 ± 5.13	74.44 ± 0.15	4.57 ± 0.01	123.25 ± 2.23	76.13 ± 0.19	1.62 ± 4.33 E-3
**Fluvastatin**	473.13 ± 2.45	85.84 ± 0.21	5.51 ± 0.01	639.93 ± 3.24	78.11 ± 0.12	8.19 ± 0.01
**Methylbenzethonium Chloride**	100 ± 2.1	70.64 ± 0.28	1.42 ± 0.01	71.46 ± 4.21	66.66 ± 0.09	1.07 ± 2.08 E-3
**Miconazole**	170.98 ± 2.41	88.14 ± 0.31	1.94 ± 0.01	130.88 ± 3.54	88.92 ± 0.08	1.47 ± 1.72 E -3
**Mycophenolic Acid**	422.4 ± 4.21	93.66 ± 0.32	4.51 ± 0.02	217.22 ± 3.41	96.66 ± 0.15	2.25 ± 3.84 E-3
**Nifuroxime**	31.61 ± 3.52	65.68 ± 0.39	0.48 ± 4.28 E-3	33.21 ± 1.43	85.1 ± 0.16	0.39 ± 9.02 E-4
**Nitroxoline**	275.28 ± 2.36	74.53 ± 0.39	3.69 ± 0.02	309.66 ± 2.56	68.44 ± 0.09	4.52 ± 0.01
**Octanoic Acid**	46.25 ± 3.21	71.7 ± 0.32	0.65 ± 3.33 E-3	36.04 ± 3.69	63.83 ± 0.86	0.56 ± 0.01
**Octodrine**	34.32 ± 2.12	96.54 ± 0.21	0.36 ± 9.93 E-4	31.61 ± 2.13	77.91 ± 0.08	0.41 ± 6.90E -4
**Pyrithione Zinc**	695.95 ± 4.21	88.19 ± 0.26	7.89 ± 0.02	553.02 ± 2.94	76.17 ± 0.12	7.26 ± 0.01
**DMSO**	39.36 ± 1.45	86.17 ± 0.17	0.46 ± 1.07 E-3	34.32 ± 1.53	104.84 ± 0.09	0.33 ± 4.27 E-4

B)

	Serum 25°C			Serum 37°C		
**Drug**	**ZOI Area (mm^2)**	**Number of Pixels**	**ZOI Area/ Number of Pixels**	**ZOI Area (mm^2)**	**Number of Pixels**	**ZOI Area/ Number of Pixels**
**Fluconazole**	39.36 ± 1.31	59.34 ± 0.09	0.66 ± 1.22 E-3	36.41 ± 1.21	85.54 ± 0.04	0.43 ± 3.40 E-4
**Antimycin**	130.08 ± 2.58	77.61 ± 0.11	1.68 ± 2.71 E-3	309.42 ± 1.45	90.16 ± 0.21	3.43 ± 8.15 E-3
**Captan**	107.75 ± 2.31	86.49 ± 0.21	1.25 ± 3.29 E-3	89.55 ± 2.41	74.94 ± 0.12	1.19 ± 2.24 E-3
**Chlorquinaldol**	148.83 ± 1.42	86.24 ± 0.18	1.73 ± 3.77 E-3	133.34 ± 1.23	75.17 ± 0.15	1.77 ± 3.70 E-3
**Clotrimazole**	52.89 ± 2.14	87.04 ± 0.04	0.61 ± 5.25 E-4	123.87 ± 1.23	76.89 ± 0.12	1.61 ± 2.67 E-3
**Disulfiram**	113.53 ± 1.54	92.54 ± 0.21	1.23 ± 2.95 E-3	118.7 ± 0.12	83.610 ± 0.09	1.42 ± 1.54 E-3
**Fluvastatin**	123.25 ± 2.52	104.75 ± 0.02	1.18 ± 4.65 E-4	68.02 ± 1.03	106.74 ± 0.04	0.64 ± 3.35 E-4
**Methylbenzethonium Chloride**	86.84 ± 1.43	84.37 ± 0.23	1.03 ± 2.98 E-3	85.55 ± 1.52	73.96 ± 0.09	1.16 ± 1.61 E-3
**Miconazole**	64.7 ± 1.53	95.27 ± 0.08	0.68 ± 7.31 E-4	55.84 ± 1.68	87.16 ± 0.12	0.64 ± 1.07 E-3
**Mycophenolic Acid**	10.21 ± 1.04	99.84 ± 0.21	0.10 ± 3.19 E-4	34.32 ± 1.24	85.38 ± 0.14	0.4 ± 8.04 E-4
**Nifuroxime**	41.08 ± 2.14	88.15 ± 0.08	0.47 ± 6.66 E-4	49.94 ± 1.76	92.34 ± 0.17	0.54 ± 1.19 E-3
**Nitroxoline**	73.8 ± 0.98	85.39 ± 0.12	0.86 ± 1.33 E-3	157.51 ± 2.41	83.71 ± 0.11	1.88 ± 4.11 E-3
**Octanoic Acid**	39.36 ± 1.23	89.45 ± 0.03	0.44 ± 2.85 E-4	45.02 ± 1.94	93.96 ± 0.09	0.48 ± 7.67 E-4
**Octodrine**	56.83 ± 1.94	85.77 ± 0.12	0.66 ± 1.15 E-3	54.25 ± 1.04	89.58 ± 0.05	0.61 ± 7.25 E-4
**Pyrithione Zinc**	27.8 ± 1.34	83.91 ± 0.05	0.33 ± 3.57 E-4	26.82 ± 0.89	63.7 ± 0.05	0.42 ± 4.70 E-4
**DMSO**	25.89 ± 1.56	89.3 ± 0.07	0.29 ± 4.02 E-4	20.173 ± 0.12	91.07 ± 0.08	0.22 ± 1.35 E-4

Supplemental Table 2: Quantification of the zone of inhibition post-treatment of various serially diluted drugs on *C. albicans* strain SN250.

	Antimycin 25°C			Antimycin 37°C		
Concentration	ZOI Area (mm^2)	Number of Pixels	ZOI Area/ Number of Pixels	ZOI Area (mm^2)	Number of Pixels	ZOI Area/ Number of Pixels
10	389.64 ± 2.95	97.05± 0.17	4.01 ± 3.61 E-3	587 ± 3.41	82.23 ± 0.31	7.14 ± 4.15 E-4
5	314.07 ± 0.25	103.05 ± 0.33	3.05 ± 0.01	501.21 ± 4.53	82.77 ± 0.31	6.06 ± 0.02
2.5	253.78 ± 1.63	114.26 ± 0.08	2.22 ± 6.56 E-3	468.46 ± 4.17	90.74 ± 0.36	5.16 ± 0.02
1.25	221.87 ± 3.70	104.09 ± 0.35	2.13 ± 1.99 E-3	423.45 ± 4.14	93.29 ± 0.34	4.54 ± 0.02
0.625	165.24 ± 1.68	91.07 ± 0.42	1.81 ± 7.16 E-3	351.61 ± 2.14	85.56 ± 0.48	4.11 ± 0.02
0.3125	158.27 ± 1.69	86.58 ± 0.52	1.83 ± 9.06 E-3	228.21 ± 4.01	72.13 ± 0.22	3.16 ± 0.02
0.15625	145.38 ± 1.41	88.69 ± 0.68	1.64 ± 9.77 E-3	202.85 ± 1.48	66.23 ± 0.52	3.06 ± 0.01
0.07812	113.05 ± 2.00	82.93 ± 0.49	1.36 ± 0.901	186.37 ± 1.14	73.72 ± 0.21	2.53 ± 0.02
DMSO	35.13 ± 0.97	93.11 ± 0.69	0.38 ± 2.86 E-3	43.81 ± 0.74	86.72 ± 0.49	0.51 ± 1.31 E-3
0.03906	66.56 ± 1.22	94.85 ± 0.77	0.7 ± 5.23 E-3	114.1 ± 1.81	89.48 ± 0.53	1.28 ± 7.19 E-3
0.01953	50.29 ± 1.52	94.18 ± 0.23	0.53 ± 4.53 E-3	81.14 ± 0.26	84.7 ± 0.18	0.96 ± 6.03 E-3
0.009765	43.612 ± 0.27	102.63 ± 0.66	0.42 ± 9.79 E-4	76.91 ± 1.31	93.56 ± 0.38	0.82 ± 1.72 E-3
0.004883	40.338 ± 0.9	100.71 ± 0.32	0.33 ± 2.71 E-3	50.29 ± 1.07	97.53 ± 0.40	0.52 ± 2.12 E-3
0.002441	31.91 ± 0.58	96.13 ± 0.37	0.33 ± 1.17 E-3	39.2 ± 0.25	102.42 ± 0.13	0.38 ± 1.52 E -3
0.00061	30.85 ± 0.45	85.52 ± 0.09	0.36 ± 1.61 E-3	35.08 ± 0.59	96.16 ± 0.24	0.36 ± 5.55 E-4
0.000305	26.2 ± 0.51	92.19 ± 0.74	0.28 ± 3.33 E-4	31.91 ± 0.36	96.37 ± 0.27	0.33 ± 8.62 E-4
0.000153	26.2 ± 0.78	98.27 ± 0.36	0.27 ± 2.09 E-3	30.85 ± 0.45	104.64 ± 0.80	0.29 ± 8.04 E-4
DMSO	26.6 ± 0.42	106.25 ± 0.47	0.25 ± 8.88E-4	35.13 ± 0.53	108.33 ± 0.09	0.32 ± 2.44 E-3

	Captan 25°C			Captan 37°C		
**Concentration**	**ZOI Area (mm^2)**	**Number of Pixels**	**ZOI Area/ Number of Pixels**	**ZOI Area (mm^2)**	**Number of Pixels**	**ZOI Area/ Number of Pixels**
10	458.11 ± 1.52	84.93 ± 0.13	5.39 ± 1.79 E-4	375.7 ± 3.12	82.48 ± 0.31	4.56 ± 3.78 E-4
5	407.29 ± 2.38	86.16 ± 0.23	4.73 ± 7.41 E-3	260.33 ± 2.13	91.68 ± 0.12	2.84 ± 9.83 E-3
2.5	337.45 ± 2.12	96.46 ± 0.32	3.5 ± 8.56 E-3	107.34 ± 2.15	72.4 ± 0.12	1.48 ± 2.75 E-3
1.25	98.47 ± 3.45	94.77 ± 0.12	1.04 ± 3.87 E-3	92.13 ± 2.12	77.82 ± 0.06	1.18 ± 2.1 E-3
0.625	72.69 ± 1.58	86.47 ± 0.12	0.84 ± 1.35E-3	84.52 ± 2.11	68.6 ± 0.12	1.23 ± 1.74 E-3
0.3125	63.39 ± 4.12	78.14 ± .23	0.81 ± 1.77 E-3	63.39 ± 2.67	60.27 ± 0.11	1.05 ± 2.54 E-3
0.15625	48.18 ± 1.95	75.3 ± 0.15	0.64 ± 2.21 E-3	45.64 ± 2.12	72.43 ± 0.04	0.63 ± 1.25 E-3
0.07812	28.95 ± 1.34	87.55 ± 0.11	0.33 ±7.20 E-4	31.91 ± 1.42	79.82 ± 0.12	0.4 ± 3.78 E-4
DMSO	37.46 ± 1.54	122.79 ± 0.11	0.31 ± 3.99 E-4	44.02 ± 1.69	150.04 ± 0.08	0.29 ± 3.47 E-4

	Chlorquinaldol 25°C			Chlorquinaldol 37°C		
**Concentration**	**ZOI Area (mm^2)**	**Number of Pixels**	**ZOI Area/ Number of Pixels**	**ZOI Area (mm^2)**	**Number of Pixels**	**ZOI Area/ Number of Pixels**
10	314.84 ± 4.12	118.01 ± .18	2.67 ± 3.49 E-4	549.81 ± 6.12	85.94 ± .12	6.4 ± 7.12 E-4
5	277.44 ± 3.12	116.52 ± .12	2.38 ± 3.95 E-3	344 ± 4.95	81.11 ± 0.24	4.24 ± 6.88 E-3
2.5	260.33 ± 3.98	119.05 ± 0.12	2.19 ± 2.54 E-3	198.2 ± 2.1	83.7 ± 0.22	2.37 ± 7.04 E-3
1.25	239.83 ± 3.1	126.48 ± 0.06	1.9 ± 2.04 E-3	191.86 ± 2.8	90.97 ± 0.13	2.11 ± 5.41 E-3
0.625	208.77 ± 2.13	111.22 ± 0.08	1.88 ± 1.2 E-3	175.8 ± 2.1	77.26 ± 0.21	2.28 ± 4.10 E-3
0.3125	158.27 ± 2.54	93.46 ± 0.12	1.69 ± 1.72 E-3	94.66 ± 2.63	66.06 ± 0.23	1.43 ± 4.95 E-3
0.15625	103.96 ± 2.12	101.97 ± 0.12	1.02 ± 1.41 E-3	32.96 ± 1.43	67.71 ± 0.12	0.49 ± 1.86 E-3
0.07812	30.85 ± 1.23	111.81 ± 0.06	0.28 ± 4.06 E-4	30.85 ± 1.21	80.98 ± 0.12	0.38 ± 7.14 E-4
DMSO	32 ± 0.67	123.24 ± 0.08	0.26 ± 1.81 E-4	31.96 ± 1.34	104.88 ± 0.11	0.3 ± 4.76 E-4

	Clotrimazole 25°C			Clotrimazole 37°C		
**Concentration**	**ZOI Area (mm^2)**	**Number of Pixels**	**ZOI Area/ Number of Pixels**	**ZOI Area (mm^2)**	**Number of Pixels**	**ZOI Area/ Number of Pixels**
10	181.93 ± 2.13	82.06 ± 0.12	2.22 ± 2.60 E-4	153.41 ± 2.14	88.79 ± 0.13	1.73 ± 2.41 E-4
5	175.38 ± 3.13	84.84 ± 0.21	2.07 ± 3.29 E -3	145.38 ± 3.56	92.97 ± 0.15	1.56 ± 2.91 E-3
2.5	165.24 ± 2.57	83.13 ± 0.12	1.99 ± 5.33 E-3	165.24 ± 3.12	98.91 ± 0.06	1.67 ± 2.85 E-3
1.25	149.6 ± 4.43	89.39 ± 0.04	1.67 ± 2.74 E-3	164.82 ± 2.12	105.39 ± 0.08	1.56 ± 1.09 E-3
0.625	145.8 ± 2.13	76.87 ± 0.03	1.9 ± 1.26 E-3	171.58 ± 3.01	85.44 ± 0.03	2.01 ± 2.23 E-3
0.3125	140.52 ± 1.23	65.72 ± 0.02	2.14 ± 1.16 E-3	141.15 ± 2.13	78.26 ± 0.09	1.8 ± 9.64 E-4
0.15625	113.05 ± 3.12	69.75 ± 0.10	1.62 ± 9.12 E-4	113.05 ± 1.43	79.76 ± 0.02	1.42 ± 1.78 E-3
0.07812	92.13 ± 2.12	71.34 ± 0.11	1.29 ± 2.11 E-3	103.33 ± 0.98	80.18 ± 0.13	1.29 ± 4.44 E-4
DMSO	37.46 ± 0.81	123.69 ± 0.04	0.3 ± 3.35 E-4	30.9 ± 2.58	95.69 ± 0.13	0.32 ± 7.08 E-4
0.03906	141.15 ± 2.13	80.66 ± 0.02	1.75 ± 1.13 E-3	108.61 ± 3.12	82.63 ± 0.15	1.31 ± 2.45 E-3
0.01953	108.61 ± 1.58	88.47 ± 0.05	1.23 ± 4.56 E-4	95.3 ± 2.12	89.72 ± 0.25	1.06 ± 2.01 E-3
0.009765	94.66 ± 1.23	87.96 ± 0.12	1.08 ± 7.52 E-4	84.1 ± 2.53	91.94 ± 0.12	0.91 ± 2.76 E-3
0.004883	81.14 ± 1.11	96.47 ± 0.08	0.84 ± 1.16 E-3	66.56 ± 1.42	96.49 ± 1.02	0.69 ± 1.01 E-3
0.002441	50.71 ± 1.47	79.27 ± 0.21	0.64 ± 8.31 E-4	45.64 ± 1.56	84.52 ± 0.93	0.54 ± 6.70 E-3
0.00061	40.57 ± 1.41	71.25 ± 0.11	0.57 ± 1.88E-3	35.08 ± 1.21	73.97 ± 0.31	0.47 ± 6.13 E-3
0.000305	37.4 ± 1.41	79.57 ± 0.05	0.47 ± 8.27 E-4	32.96 ± 1.94	76.95 ± 0.11	0.43 ± 1.98 E-3
0.000153	32.96 ± 1.03	86.18 ± 0.89	0.38 ± 3.41 E-4	23.67 ± 1.03	86.21 ± 0.84	0.27 ± 4.7 E-4
DMSO	34.71 ± 1.76	80.82 ± 0.12	0.43 ± 4.95 E-3	33.02 ± 1.42	92.14 ± 0.62	0.36 ± 3.42 E-3

	Disulfiram 25°C			Disulfiram 37°C		
**Concentration**	**ZOI Area (mm^2)**	**Number of Pixels**	**ZOI Area/ Number of Pixels**	**ZOI Area (mm^2)**	**Number of Pixels**	**ZOI Area/ Number of Pixels**
10	181.72 ± 3.21	64.79 ± 0.21	2.8 ± 4.95 E-4	103.33 ± 1.32	76.23 ± 0.44	1.36 ± 1.73 E-4
5	150.24 ± 2.12	70.57 ± 0.23	2.13 ± 6.64 E-3	35.08 ± 1.11	71.66 ± 0.21	0.49 ± 3.16 E-3
2.5	40.57 ± 1.64	82.34 ± 0.21	0.49 ± 1.58 E-3	34.65 ± 1.33	83.49 ± 0.05	0.42 ± 1.20 E-3
1.25	37.19 ± 1.54	74.6 ± 0.24	0.5 ± 1.61 E-3	35.08 ± 0.08	99.74 ± 0.21	0.35 ± 1.84 E-4
0.625	36.34 ± 1.08	84.62 ± 0.07	0.43 ± 1.35 E-3	32.96 ± 0.92	65.73 ± 0.13	0.5 ± 1.74 E-3
0.3125	35.08 ± 2.42	60.39 ± 0.07	0.58 ± 1.07 E-3	28.1 ± 0.87	76.44 ± 0.23	0.37 ± 7.39 E-4
0.15625	29.58 ± 2.09	63.93 ± 0.09	0.46 ± 8.34 E-4	32.96 ± 1.21	75.73 ± 0.18	0.44 ± 1.48 E-3
0.07812	28.74 ± 1.03	82.05 ± 0.05	0.35 ± 5.10 E-4	31.27 ± 0.85	90.23 ± 0.23	0.35 ± 7.86 E-4
DMSO	32.02 ± 0.78	95 ± 0.12	0.34 ± 2.60 E-4	33.02 ± 0.68	100.6 ± 0.15	0.33 ± 8.18 E-4

	Fluconazole 25°C			Fluconazole 37°C		
**Concentration**	**ZOI Area (mm^2)**	**Number of Pixels**	**ZOI Area/ Number of Pixels**	**ZOI Area (mm^2)**	**Number of Pixels**	**ZOI Area/ Number of Pixels**
10	626.73 ± 5.12	88.91 ± 0.14	7.05 ± 5.76 E-4	721.6 ± 5.12	97.31 ± 0.07	7.42 ± 5.26 E-4
5	491.49 ± 4.21	95.02 ± 0.44	5.17 ± 8.06 E-3	494.03 ± 6.12	100.2 ± 0.12	4.93 ± 4.06 E-3
2.5	406.55 ± 4.82	106.75 ± 0.32	3.81 ± 1.61 E-2	314.84 ± 3.59	113.9 ± 0.32	2.76 ± 3.23 E-3
1.25	375.28 ± 3.16	107.25 ± 0.08	3.5 ± 1.07 E-2	216.16 ± 3.21	112.1 ± 0.18	1.93 ± 5.79 E-3
0.625	215.53 ± 1.25	107.53 ± 0.46	2 ± 1.61 E-3	141.15 ± 1.69	93.67 ± 0.13	1.51 ± 3.08 E-3
0.3125	81.14 ± 1.05	97.21 ± 0.08	0.83 ± 4.06 E-3	63.39 ± 1.42	86.84 ± 0.29	0.73 ± 1.31 E-3
0.15625	30.85 ± 0.54	97.07 ± 0.03	0.32 ± 3.18 E-4	48.39 ± 0.98	107.39 ± 0.22	0.45 ± 2.57 E-3
0.07812	31.91 ± 1.57	122.23 ± 0.06	0.26 ± 1.93 E-4	40.57 ± 1.95	98.51 ± 0.08	0.41 ± 1.12 E-3
DMSO	37.25 ± 0.96	119.78 ± 0.04	0.31 ± 2.36 E-4	39.46 ± 1.42	115.93 ± 0.06	0.34 ± 3.57 E-4

	Fluvastatin 25°C			Fluvastatin 37°C		
**Concentration**	**ZOI Area (mm^2)**	**Number of Pixels**	**ZOI Area/ Number of Pixels**	**ZOI Area (mm^2)**	**Number of Pixels**	**ZOI Area/ Number of Pixels**
10	48.18 ± 2.13	72.52 ± 0.03	0.66 ± 2.94 E-4	484.31 ± 4.42	72.55 ± 0.04	6.68 ± 6.09 E-4
5	43.95 ± 2.49	74.25 ± 0.02	0.59 ± 5.75 E-4	407.82 ± 3.42	82.28 ± 0.14	4.96 ± 2.83 E-3
2.5	37.4 ± 1.23	81.88 ± 0.05	0.46 ± 2.62 E-4	381.19 ± 2.43	85.75 ± 0.21	4.45 ± 0.01
1.25	43.95 ± 0.93	86.93 ± 0.13	0.51 ± 3.98 E-4	286.11 ± 1.23	89.35 ± 0.11	3.2 ± 7.66 E-3
0.625	48.18 ± 1.42	96.48 ± 0.21	0.5 ± 8.20 E-4	216.16 ± 2.41	75.34 ± 0.06	2.87 ± 4.51 E-3
0.3125	45.64 ± 0.34	71.42 ± 0.31	0.64 ± 1.93 E-3	186.37 ± 2.65	68.63 ± 0.32	2.72 ± 2.76 E-3
0.15625	35.08 ± 0.13	75.21 ± 0.12	0.47 ± 1.94 E-3	129.11 ± 1.69	69.07 ± 0.31	1.87 ± 8.90 E-3
0.07812	31.91 ± 0.08	74.05 ± 0.21	0.43 ± 7.09 E-4	129.92 ± 3.21	67.74 ± 0.12	1.92 ± 9.25 E-3
DMSO	33.62 ± 0.12	75.64 ± 0.07	0.44 ± 1.25 E-3	28.99 ± 1.02	93.59 ± 0.12	0.31 ± 5.06 E-4

	Methylbenzethonium Chloride 25°C			Methylbenzethonium Chloride 37°C		
**Concentration**	**ZOI Area (mm^2)**	**Number of Pixels**	**ZOI Area/ Number of Pixels**	**ZOI Area (mm^2)**	**Number of Pixels**	**ZOI Area/ Number of Pixels**
10	121.71 ± 1.46	61.23 ± 0.12	1.99 ± 2.38 E-4	92.13 ± 1.04	69.39 ± 0.02	1.33 ± 1.5 E-4
5	99.74 ± 0.59	60.78 ± 0.06	1.64 ± 3.34 E-3	80.3 ± 1.41	76.06 ± 0.08	1.06 ± 4.63 E-4
2.5	66.56 ± 0.74	63.06 ± 0.21	1.06 ± 1.12 E-3	57.9 ± 1.32	68.63 ± 0.13	0.84 ± 1.18 E-3
1.25	45.64 ± 0.84	66.26 ± 0.19	0.69 ± 2.31 E-3	47.54 ± 1.09	72.52 ± 0.14	0.66 ± 1.33 E-3
0.625	30.85 ± 1.04	82.81 ± 0.09	0.37 ± 9.80 E-4	44.37 ± 0.98	84.45 ± 0.05	0.53 ± 9.87 E-4
0.3125	31.91 ± 0.23	77.26 ± 0.08	0.41 ± 5.11 E-4	29.58 ± 0.78	82.05 ± 0.07	0.36 ± 3.15 E-4
0.15625	30.85 ± 0.95	71.97 ± 0.02	0.43 ± 6.08 E-4	30.85 ± 0.19	89.59 ± 0.12	0.34 ± 2.9 E-4
0.07812	31.91 ± 0.53	86.09 ± 0.09	0.37 ± 1.48 E-4	24.3 ± 1.32	100.09 ± 0.08	0.24 ± 4.23 E-4
DMSO	35.13 ± 0.23	93.16 ± 0.09	0.38 ± 3.89 E-4	31.96 ± 1.03	102.71 ± 0.04	0.31 ± 3.43 E-4

	Miconazole 25°C			Miconazole 37°C		
**Concentration**	**ZOI Area (mm^2)**	**Number of Pixels**	**ZOI Area/ Number of Pixels**	**ZOI Area (mm^2)**	**Number of Pixels**	**ZOI Area/ Number of Pixels**
10	108.61 ± 1.32	46.07 ± 0.03	2.36 ± 2.87 E-4	108.61 ± 5.48	81.88 ± 0.02	1.33 ± 6.69 E-4
5	92.13 ± 1.43	48.82 ± 0.12	1.89 ± 1.45 E-3	99.74 ± 1.32	87.44 ± 0.01	1.14 ± 4.12 E-4
2.5	84.52 ± 1.49	59.34 ± 0.14	1.42 ± 3.13 E-3	91.71 ± 1.43	89.97 ± 0.12	1.02 ± 2.72 E-4
1.25	88.96 ± 1.46	60.83 ± 0.08	1.46 ± 3.61 E-3	91.71 ± 1.23	90.9 ± 0.09	1.01 ± 1.47 E-3
0.625	84.1 ± 1.31	65.02 ± 0.04	1.29 ± 1.79 E-3	95.3 ± 0.98	90.82 ± 0.21	1.05 ± 1.15 E-3
0.3125	80.93 ± 1.23	57.31 ± 0.23	1.41 ± 1.20 E-3	84.52 ± 0.84	73.78 ± 0.18	1.15 ± 3.37 E-3
0.15625	73.75 ± 0.90	54.9 ± 0.21	1.34 ± 5.79 E-3	73.75 ± 1.29	71.06 ± 0.14	1.04 ± 2.81 E-3
0.07812	66.77 ± 0.08	52.01 ± 0.12	1.28 ± 5.20 E-3	54.09 ± 1.04	87.27 ± 0.21	0.62 ± 1.11 E-3
DMSO	35.13 ± 0.58	63.24 ± 0.18	0.56 ± 1.15 E-3	26.24 ± 1.03	87.03 ± 0.08	0.3 ± 8.46 E-4
0.03906	73.75 ± 1.53	92.62 ± 0.03	0.8 ± 1.71 E-3	47.54 ± 1.81	94.41 ± 0.03	0.5 ± 6.18 E-4
0.01953	69.73 ± 2.08	96.8 ± 0.09	0.72 ± 4.38 E-4	39.3 ± 1.18	107.03 ± 0.18	0.37 ± 2.13 E-4
0.009765	56.63 ± 1.74	102.95 ± 0.14	0.55 ± 6.50 E-4	27.05 ± 1.09	113.44 ± 0.09	0.24 ± 4.74 E-4
0.004883	55.78 ± 1.43	110.23 ± 0.05	0.51 ± 7.72 E-4	20.5 ± 1.03	122.95 ± 0.07	0.17 ± 2.06 E-4
0.002441	43.74 ± 1.48	113.07 ± 0.03	0.39 ± 3.02 E-4	21.55 ± 0.93	124.48 ± 0.03	0.17 ± 1.72 E-4
0.00061	30.85 ± 1.85	97.49 ± 0.04	0.32 ± 2.87 E-4	30.85 ± 0.83	103.51 ± 0.11	0.3 ± 1.67 E-4
0.000305	28.1 ± 0.94	99.35 ± 0.07	0.28 ± 2.08 E-4	18.6 ± 1.21	93.57 ± 0.12	0.2 ± 3.63 E-4
0.000153	31.91 ± 1.34	103.46 ± 0.14	0.31 ± 3.38 E-4	28.95 ± 1.04	106.1 ± 0.08	0.27 ± 4.07 E-4
DMSO	30.9 ± 1.48	112.13 ± 0.09	0.28 ± 4.76 E-4	24.34 ± 1.02	111.68 ± 0.19	0.22 ± 2.47 E-4

	Mycophenolic Acid 25°C			Mycophenolic Acid 37°C		
**Concentration**	**ZOI Area (mm^2)**	**Number of Pixels**	**ZOI Area/ Number of Pixels**	**ZOI Area (mm^2)**	**Number of Pixels**	**ZOI Area/ Number of Pixels**
10	300.47 ± 4.12	116.81 ± 0.13	2.57 ± 3.53 E-4	468.46 ± 5.43	105.77 ± 0.02	4.43 ± 5.13 E-4
5	266.24 ± 3.14	106.15 ± 0.21	2.51 ± 3.37 E-3	202.85 ± 4.23	109.95 ± 0.04	1.84 ± 7.20 E-4
2.5	159.11 ± 1.94	116.01 ± 0.09	1.37 ± 2.65 E-3	113.05 ± 1.40	118.06 ± 0.03	0.96 ± 4.43 E-4
1.25	47.54 ± 0.58	116.17 ± 0.10	0.41 ± 3.67 E-4	37.4 ± 1.23	119.94 ± 0.12	0.31 ± 1.81 E-4
0.625	31.91 ± 0.64	109.13 ± 0.13	0.29 ± 3.27 E-4	16.69 ± 1.09	103.46 ± 0.04	0.16 ± 2.92 E-4
0.3125	45.64 ± 1.74	96.83 ± 0.04	0.47 ± 8.13 E-4	21.98 ± 1.02	87.21 ± 0.13	0.25 ± 2.33 E-4
0.15625	32.96 ± 0.65	89.3 ± 0.09	0.37 ± 2.38 E-4	26.2 ± 0.98	90.82 ± 0.05	0.29 ± 5.21 E-4
0.07812	32.96 ± 0.98	99.35 ± 0.08	0.33 ± 3.99 E-4	30.85 ± 0.87	106.51 ± 0.02	0.29 ± 2.18 E-4
DMSO	37.46 ± 1.23	115.57 ± 0.19	0.32 ± 3.31 E-4	30.9 ± 1.21	116.85 ± 0.08	0.26 ± 1.49 E-4

	Nifuroxime 25°C			Nifuroxime 37°C		
**Concentration**	**ZOI Area (mm^2)**	**Number of Pixels**	**ZOI Area/ Number of Pixels**	**ZOI Area (mm^2)**	**Number of Pixels**	**ZOI Area/ Number of Pixels**
10	117.91 ± 1.21	82.44 ± 0.04	1.43 ± 1.47 E-4	30.85 ± 2.31	68.18 ± 0.05	0.45 ± 3.39 E-4
5	37.19 ± 1.48	85.47 ± 0.09	0.44 ± 3.77 E-4	28.95 ± 2.03	91.1 ± 0.02	0.32 ± 3.97 E-4
2.5	26.2 ± 1.25	86.26 ± 0.14	0.3 ± 4.62 E-4	30.85 ± 1.24	91.86 ± 0.08	0.34 ± 2.08 E-4
1.25	35.08 ± 1.02	106.37 ± 0.21	0.33 ± 5.30 E-4	28.95 ± 1.49	97.82 ± 0.21	0.3 ± 3.94 E-4
0.625	31.91 ± 1.92	77.87 ± 0.16	0.41 ± 1.35 E-3	37.4 ± 1.09	90.09 ± 0.06	0.42 ± 1.09 E-3
0.3125	37.4 ± 1.32	79.83 ± 0.04	0.47 ± 1.10 E-3	37.19 ± 1.24	72.98 ± 0.12	0.51 ± 5.89 E-4
0.15625	32.96 ± 1.25	72.67 ± 0.21	0.45 ± 4.22 E-4	28.95 ± 0.78	79.51 ± 0.08	0.36 ± 6.58 E-4
0.07812	31.91 ± 0.89	85.06 ± 0.09	0.38 ± 1.03 E-3	26.2 ± 1.13	88.64 ± 0.08	0.3 ± 3.94 E-4
DMSO	37.46 ± 0.92	98.91 ± 0.11	0.38 ± 4.38 E-4	47.62 ± 1.04	99.82 ± 0.23	0.48 ± 4.87 E-4

	Nitroxoline 25°C			Nitroxoline 37°C		
**Concentration**	**ZOI Area (mm²)**	**Number of Pixels**	**ZOI Area/ Number of Pixels**	**ZOI Area (mm²)**	**Number of Pixels**	**ZOI Area/ Number of Pixels**
10	228.21 ± 4.12	66.95 ± 0.08	3.41 ± 6.15 E4	286.11 ± 5.31	73.19 ± 0.04	3.91 ± 7.26 E-4
5	210.04 ± 3.18	78.06 ± 0.12	2.69 ± 3.17 E-3	245.53 ± 3.12	68.46 ± 0.12	3.59 ± 2.55 E-3
2.5	181.93 ± 1.21	85.43 ± 0.21	2.13 ± 3.13 E-3	222.29 ± 4.12	80.96 ± 0.03	2.75 ± 4.58 E-3
1.25	121.92 ± 1.97	97.69 ± 0.04	1.25 ± 2.88 E-3	143.69 ± 1.92	96.8 ± 0.21	1.48 ± 6.58 E-4
0.625	50.29 ± 0.92	82.99 ± 0.12	0.61 ± 4.03 E-4	81.14 ± 1.84	89.71 ± 0.12	0.9 ± 2.32 E-3
0.3125	40.57 ± 1.23	74.42 ± 0.06	0.55 ± 1.04 E-3	31.91 ± 0.98	83.96 ± 0.23	0.38 ± 6.60 E-4
0.15625	37.19 ± 1.03	88.76 ± 0.13	0.42 ± 3.99 E-4	29.58 ± 0.83	94.99 ± 0.14	0.31 ± 8.41 E-4
0.07812	32.96 ± 1.08	96.82 ± 0.09	0.34 ± 5.69 E-4	30.85 ± 0.91	103.98 ± 0.13	0.3 ± 4.87 E-4
DMSO	45.72 ± 2.01	97.98 ± 0.17	0.47 ± 6.34 E-4	32.9 ± 1.03	89.64 ± 0.14	0.37 ± 6.47 E-4

	Octanoic Acid 25°C			Octanoic Acid 37°C		
**Concentration**	**ZOI Area (mm²)**	**Number of Pixels**	**ZOI Area/ Number of Pixels**	**ZOI Area (mm²)**	**Number of Pixels**	**ZOI Area/ Number of Pixels**
10	181.93 ± 2.31	72.29 ± 0.12	2.52 ± 3.20 E-4	31.91 ± 2.03	55.49 ± 0.12	0.58 ± 3.66 E-4
5	170.52 ± 3.45	72.46 ± 0.21	2.35 ± 4.37 E-3	28.74 ± 1.93	69.99 ± 0.11	0.41 ± 9.80 E-4
2.5	129.11 ± 1.85	79.52 ± 0.19	1.62 ± 4.52 E-3	26.2 ± 0.23	87.76 ± 0.12	0.3 ± 4.00 E-4
1.25	91.71 ± 3.05	90.09 ± 0.09	1.02 ± 2.49 E-3	21.98 ± 0.45	100.89 ± 0.03	0.22 ± 3.04 E-4
0.625	66.77 ± 2.58	83.24 ± 0.19	0.8 ± 1.18 E-3	18.17 ± 1.25	105.58 ± 0.05	0.17 ± 1.67 E-4
0.3125	45.64 ± 2.12	74 ± 0.29	0.62 ± 1.87 E-3	14.58 ± 1.02	93.09 ± 0.09	0.16 ± 1.94 E-4
0.15625	37.19 ± 2.90	74.07 ± 0.19	0.5 ± 2.36 E-3	35.08 ± 1.08	94.7 ± 0.12	0.37 ± 6.37 E-4
0.07812	24.3 ± 1.76	74.36 ± 0.29	0.33 ± 1.07 E-3	35.08 ± 1.63	95.1 ± 0.15	0.37 ± 6.37 E-4
DMSO	25 ± 1.07	99.66 ± 0.12	0.25 ± 8.37 E-4	30.9 ± 1.25	97.67 ± 0.19	0.32 ± 6.14 E-4
0.03906	35.08 ± 1.04	77.19 ± 0.07	0.45 ± 8.41 E-4	30.85 ± 1.20	91.86 ± 0.09	0.34 ± 8.25 E-4
0.01953	32.96 ± 1.29	99.15 ± 0.12	0.33 ± 3.65 E-4	30.85 ± 1.23	105.08 ± 0.09	0.29 ± 3.69 E-4
0.009765	35.08 ± 1.05	107.15 ± 0.32	0.33 ± 4.65 E-4	29.58 ± 1.40	113.21 ± 0.08	0.26 ± 3.31 E-4
0.004883	23.67 ± 2.05	104.66 ± 0.19	0.23 ± 8.87 E-4	14.79 ± 1.13	110.61 ± 0.08	0.13 ± 1.99 E-4
0.002441	26.2 ± 1.67	103.78 ± 0.04	0.25 ± 6.23 E-4	-	-	-
0.00061	23.67 ± 2.39	96.36 ± 0.12	0.25 ± 3.50 E-4	31.91 ± 1.64	77.9 ± 0.12	0.41 ± 2.11 E-4
0.000305	23.67 ± 3.12	89.45 ± 0.12	0.26 ± 7.04 E-4	28.74 ± 1.41	80.12 ± 0.07	0.36 ± 7.13 E-4
0.000153	23.67 ± 1.28	90.36 ± 0.09	0.26 ± 4.90 E-4	29.58 ± 2.20	93.09 ± 0.12	0.32 ± 4.75 E-4
DMSO	27.15 ± 1.08	106.06 ± 0.11	0.26 ± 3.19 E-4	22.01 ± 1.23	84.94 ± 0.08	0.26 ± 5.11 E-4

	Octodrine 25°C			Octodrine 37°C		
**Concentration**	**ZOI Area (mm²)**	**Number of Pixels**	**ZOI Area/ Number of Pixels**	**ZOI Area (mm²)**	**Number of Pixels**	**ZOI Area/ Number of Pixels**
10	35.08 ± 1.32	104.46 ± 0.08	0.34 ± 1.26 E-4	30.23 ± 2.13	98.27 ± 0.03	0.31 ± 2.17 E-4
5	27.27 ± 1.53	116.03 ± 0.09	0.24 ± 2.94 E-4	23.02 ± 1.94	111.36 ± 0.12	0.21 ± 2.30 E-4
2.5	28.95 ± 2.15	117.33 ± 0.14	0.25 ± 3.73 E-4	22.61 ± 1.84	127.7 ± 0.09	0.18 ± 3.10 E-4
1.25	29.23 ± 1.03	112 ± 0.18	0.26 ± 4.18 E-4	21.98 ± 1.59	113.1 ± 0.07	0.19 ± 2.95 E-4
0.625	28.74 ± 0.94	103.63 ± 0.17	0.28 ± 5.72 E-4	21.98 ± 1.94	108.63 ± 0.05	0.2 ± 3.09 E-4
0.3125	29.2 ± 1.25	91.87 ± 0.14	0.32 ± 7.24 E-4	20.82 ± 0.93	97.61 ± 0.13	0.21 ± 2.05 E-4
0.15625	28.1 ± 1.04	99.05 ± 0.18	0.28 ± 5.06 E-4	24.3 ± 1.31	101.79 ± 0.08	0.24 ± 4.34 E-4
0.07812	27.99 ± 1.23	101.65 ± 0.13	0.27 ± 6.09 E-4	26.2 ± 1.09	96.5 ± 0.09	0.27 ± 3.38 E-4
DMSO	24.82 ± 1.34	106.7 ± 0.09	0.23 ± 4.09 E-4	27.23 ± 1.32	104.64 ± 0.12	0.26 ± 3.50 E-4

	Pyrithione Zinc 25°C			Pyrithione Zinc 37°C		
**Concentration**	**ZOI Area (mm²)**	**Number of Pixels**	**ZOI Area/ Number of Pixels**	**ZOI Area (mm²)**	**Number of Pixels**	**ZOI Area/ Number of Pixels**
10	682.09 ± 6.13	99.8 ± 0.23	6.83 ± 6.14 E-4	587 ± 4.12	80.54 ± 0.36	7.29 ± 5.12 E-4
5	390.07 ± 4.12	96.65 ± 0.08	4.04 ± 0.01	425.57 ± 5.68	74.46 ± 0.43	5.72 ± 0.03
2.5	273 ± 3.31	99.11 ± 0.03	2.75 ± 2.56 E-3	415.85 ± 3.58	86.34 ± 0.22	4.82 ± 0.02
1.25	237.51 ± 4.52	111.49 ± 0.12	2.13 ± 9.79 E-4	264.34 ± 1.65	91.79 ± 0.32	2.88 ± 7.08 E-3
0.625	273 ± 5.23	104.09 ± 0.22	2.62 ± 3.53 E-3	229.26 ± 1.05	82.8 ± 0.12	2.77 ± 0.01
0.3125	233.28 ± 2.56	86.48 ± 0.18	2.7 ± 7.16 E-3	45.64 ± 0.98	69.1 ± 0.22	0.66 ± 1.29 E-3
0.15625	145.8 ± 2.41	89.65 ± 0.21	1.63 ± 3.53 E-3	40.57 ± 1.20	67.71 ± 0.08	0.6 ± 2.12 E-3
0.07812	37.4 ± 1.98	92.2 ± 0.11	0.41 ± 1.14 E-3	37.19 ± 1.09	85.09 ± 0.09	0.44 ± 5.39 E-4
DMSO	40.64 ± 2.14	114.84 ± 0.08	0.35 ± 5.25 E-4	5.5 ± 0.68	112.27 ± 0.06	0.05 ± 9.98 E-5

Supplemental Table 3: Serial dilution experiments of Octodrine on serum and YPD plates

	Serum 37°C			YPD 37°C		
**Concentration**	**ZOI Area (mm^2)**	**Number of Pixels**	**ZOI Area/ Number of Pixels**	**ZOI Area (mm^2)**	**Number of Pixels**	**ZOI Area/ Number of Pixels**
6000 mM (Neat)	2593.32 ± 6.21	113.67 ± 0.12	22.81 ± 5.46 E-4	1154.21 ± 2.31	71.29 ± 0.43	16.19 ± 3.24 E-4
3000 mM	914.11 ± 4.22	103.23 ± 0.32	8.86 ± 0.01	545.84 ± 2.54	76.92 ± 0.23	7.10 ± 0.04
1500 mM	635.61 ± 2.31	85.25 ± 0.32	7.46 ± 0.03	164.23 ± 3.24	74.12 ± 0.42	2.22 ± 7.31 E-3
750 mM	378.21 ± 3.12	98.45 ± 0.32	3.84 ± 0.01	61.24 ± 1.21	76.78 ± 0.03	0.80 ± 4.52 E-3
375 mM	109.83 ± 1.41	90.42 ± 0.23	1.21 ± 4.45 E-3	28.32 ± 2.12	64.23 ± 0.32	0.44 ± 5.36 E-4
187.50 mM	73.83 ± 1.12	91.43 ± 0.53	0.81 ± 2.15 E-3	26.12 ± 1.23	59.32 ± 0.21	0.44 ± 2.58 E-3
93.75 mM	54.55 ± 2.12	71.52 ± 0.15	0.76 ± 5.95 E-3	27.98 ± 2.31	60.21 ± 0.23	0.46 ± 2.00 E-3
46.88 mM	40.1 ± 2.12	75.23 ± 0.21	0.53 ± 1.34 E-3	26.94 ± 2.53	61.32 ± 0.23	0.44 ± 2.06 E-3
23.44 mM	20.29 ± 1.32	83.98 ± 0.34	0.24 ± 7.61 E-4	23.41 ± 1.23	60.12 ± 0.23	0.39 ± 1.69 E-3
11.72 mM	24.12 ± 2.32	84.21 ± 0.25	0.29 ± 1.43 E-3	22.41 ± 2.31	62.43 ± 0.37	0.36 ± 3.70 E-4
5.86 mM	26.21 ± 2.56	79.21 ± 0.32	0.33 ± 1.37 E-3	20.31 ± 1.25	57.23 ± 0.21	0.35 ± 2.51 E-3
2.30 mM	22.58 ± 1.42	73.92 ± 0.23	0.31 ± 1.51 E-3	18.33 ± 2.31	65.21 ± 0.32	0.28 ± 1.26 E-3
DMSO	-	-	-	16.23 ± 0.21	62.12 ± 0.32	0.26 ± 3.38 E-5

Supplemental Table 4: Quantification of the zone of inhibition of Octodrine in neat form against *Candida albicans*, *Escherichia coli and Bacillus cereus.*

Species	ZOI Area (mm^2)	Number of Pixels	ZOI Area/Number of Pixels
**Candida albicans**	2852.79 ± 8.31	86.48 ± 0.35	32.99 ± 9.61 E-4
**Bacillus cereus**	329.92 ± 7.31	58.54 ± 0.24	5.64 ± 0.03
**Escherichia coli**	764.11 ± 8.45	70.34 ± 0.23	10.86 ± 0.04

Supplemental Figure 1: Elucidation of *Candida albicans* sensitivity to Octodrine

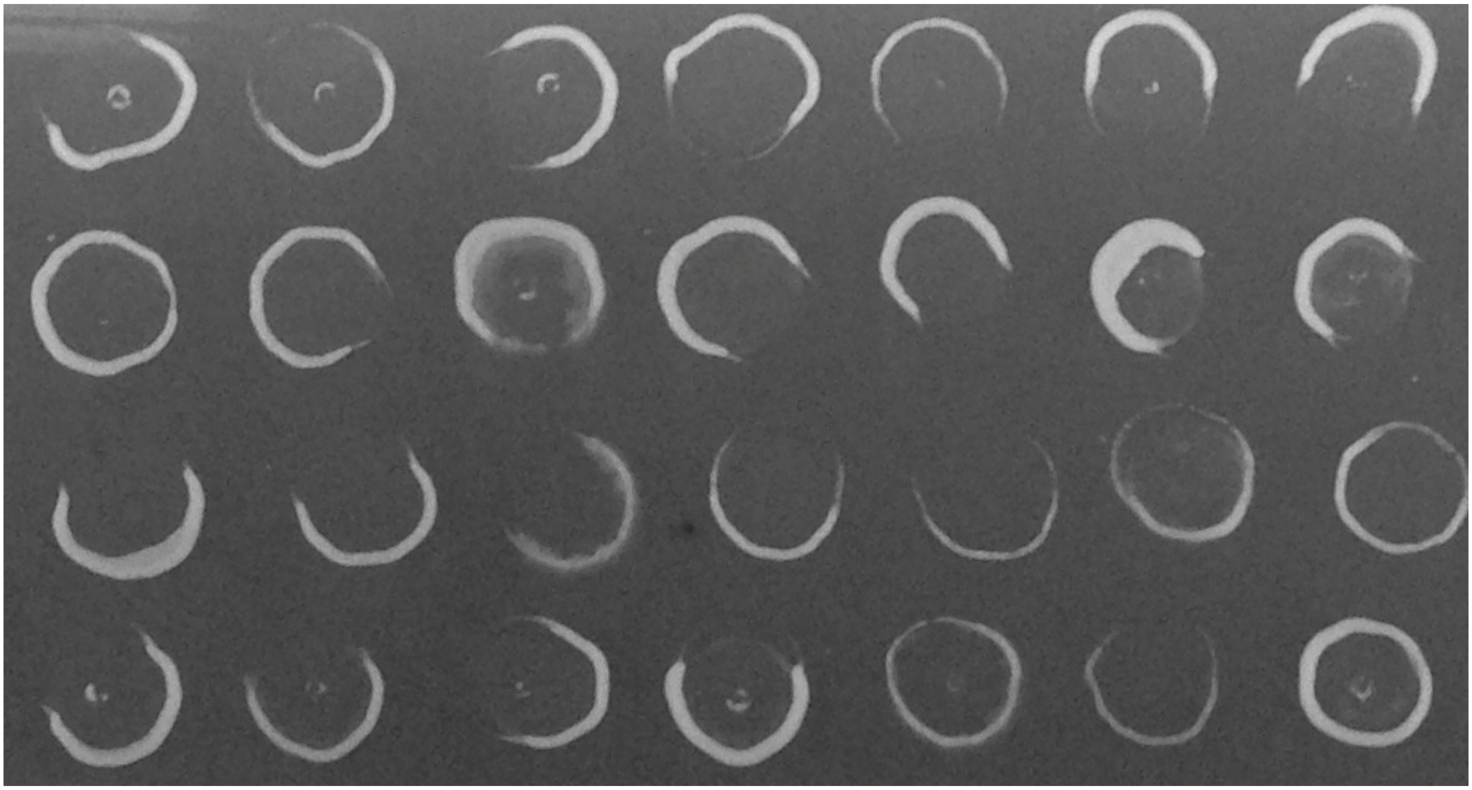

Supplement: Additional file 1: — Quantification of the zone of inhibition post-treatment of various drugs on C. albicans strain SN250. Quantification of the zone of inhibition post-treatment of various serially diluted drugs on C. albicans strain SN250. Serial dilution experiments of Octodrine on serum and YPD plates. Quantification of the zone of inhibition of Octodrine in neat form against Candida albicans, Escherichia coli and Bacillus cereus. Elucidation of mechanism of Candida albicans: Elucidation of Candida albicans sensitivity to Octodrine. [file 12941_2015_90_MOESM1_ESM.pdf]
